# Antioxidant and Preservative Effects of *Epilobium angustifolium* Extract in Beef Burgers Products: Physicochemical Properties, Color Stability, Lipid Oxidation, and Molecular Docking Analyses

**DOI:** 10.1002/fsn3.70125

**Published:** 2025-03-26

**Authors:** Nazik Meziyet Dilek, Abidin Gümrükçüoğlu, Gamze Demirel, Alper Durmaz, Emine Incilay Torunoğlu, Erdi Can Aytar, Kübra Ünal

**Affiliations:** ^1^ Akşehir Kadir Yallagöz School of Health—Department of Nutrition and Dietetics Selçuk University Konya Turkey; ^2^ Medicinal‐Aromatic Plants Application and Research Center Artvin Çoruh University Artvin Turkey; ^3^ Ali Nihat Gökyiğit Botanical Garden Application and Research Center Artvin Çoruh University Artvin Turkey; ^4^ Faculty of Medicine, Department of Medical Biochemistry Necmettin Erbakan University Konya Turkey; ^5^ Faculty of Agriculture, Department of Horticulture Usak University Uşak Turkey; ^6^ Faculty of Agriculture, Department of Food Engineering Selçuk University Konya Turkey

**Keywords:** *Epilobium angustifolium*, GC–MS analysis, HPLC analysis, molecular docking, TBARS analysis

## Abstract

Consumers are increasingly seeking natural alternatives to synthetic preservatives in meat products. This study evaluated the effects of willow *E. angustifolium* extract on beef burgers' physicochemical properties, color stability, and lipid oxidation during refrigerated storage. The extract demonstrated significant antioxidant activity, with a total phenolic content of 1263.48 mg GAE/L and a total flavonoid content of 278.43 mg ce/L. The pH values of the beef burgers decreased over time, with significant effects on Days 4 and 8 in the treated groups, except T1. The color properties, including lightness (L*), redness (a*), and yellowness (b*), were affected by the plant extract, with 1 g and 3 g concentrations leading to darker and redder hues. Moreover, adding 9 g extract led to discoloration due to increased b* values. Thiobarbituric acid‐reactive substances analysis showed increased lipid oxidation, with the T3 group (9 g extract) exhibiting higher values, suggesting a prooxidant effect at higher concentrations. Gas chromatography–mass spectrometry analysis identified a range of phytochemicals, including α‐pinene and β‐thujone, which contributed to the complex, volatile profile of the extract. High‐performance liquid chromatography analysis revealed the presence of significant phenolic compounds, such as ascorbic acid and gallic acid, with high antioxidant potential. Molecular docking studies indicated that gallic acid exhibited a moderate binding affinity with the target protein 9R‐lipoxygenase (5EK8), followed by β‐thujone and α‐pinene, suggesting their potential as bioactive compounds in food preservation. The results provide insights into the functional potential of *E. angustifolium* extract as a natural antioxidant and preservative in meat products.

## Graphical abstract

This study evaluates the antioxidant and preservative effects of *Epilobium angustifolium* extract in beef burgers during refrigerated storage. The extract influenced physicochemical properties, color stability, and lipid oxidation, with higher concentrations (900 ppm) exhibiting a prooxidant effect. Molecular docking analysis highlighted gallic acid, β‐thujone, and α‐pinene as key bioactive compounds, supporting the extract's potential as a natural meat preservative.

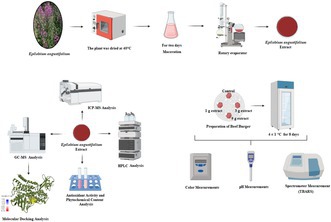

**Abbreviations**
Å: Ångström
DPPH: 2,2‐diphenyl‐1‐picrylhydrazyl
FQ: fluoroquinolone
FRAP: ferric reducing antioxidant power
GC–MS: gas chromatography–mass spectrometry
HPLC: high‐performance liquid chromatography
K: potassium
Ki: inhibition constant
LE: lipoid extract
Mg: magnesium
*p*IC_50_
: half maximal inhibitory concentration
ppm: parts per million
TBARS: thiobarbituric acid reactive substances
TFC: total flavonoid content
TPC: total phenolic content

## 1 Introduction

**FIGURE 1:**
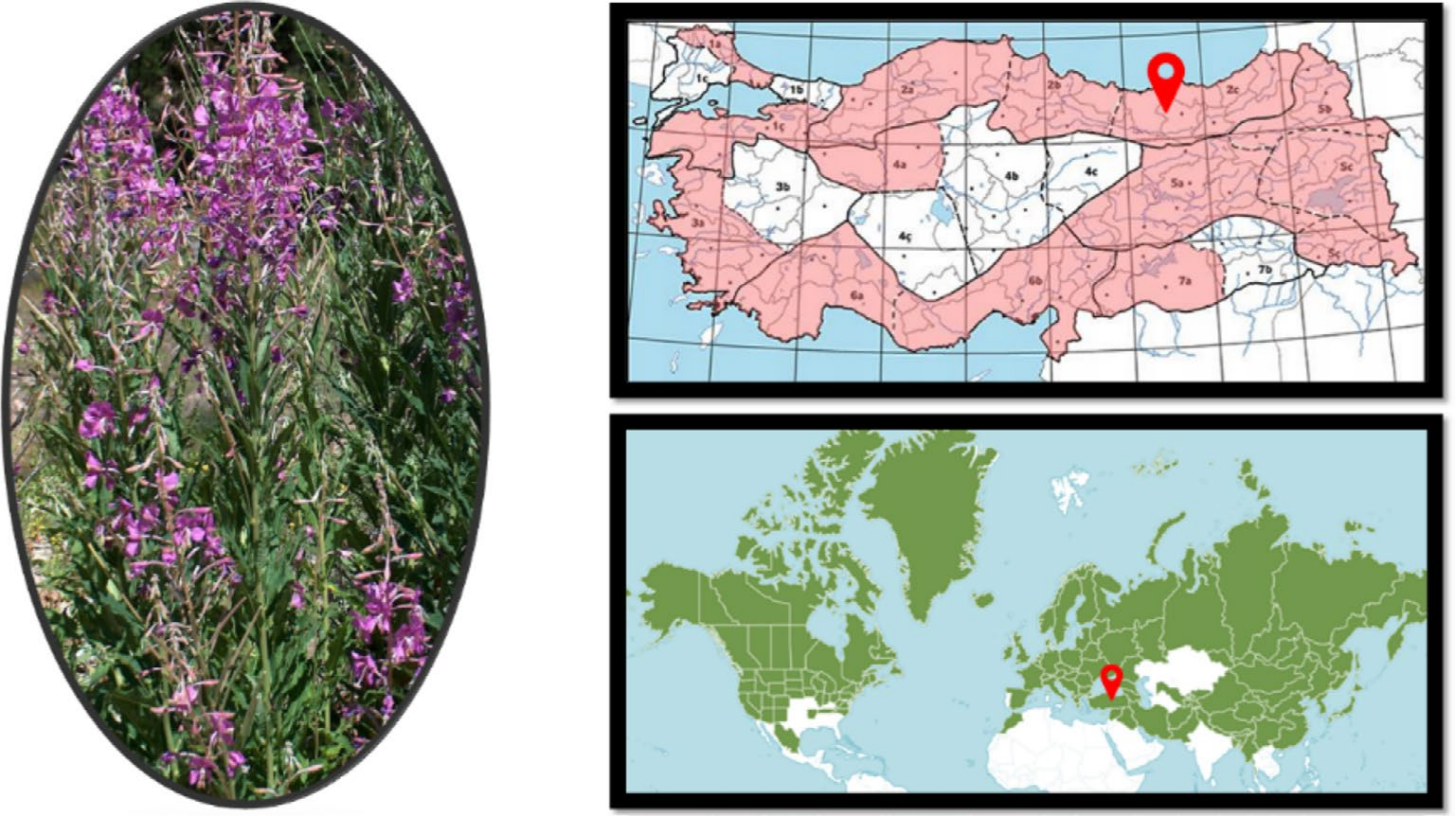
Biogeography, distribution, and collection site of *Epilobium angustifolium*.

*Epilobium angustifolium* L. in sp. pl.: 347 (1753) (Onagraceae) (syn. *Chamerion angustifolium* (L.) Holub, *Chamaenerion angustifolium* (L.) Scop.) (Figure 1) is one of the 189 globally accepted species of the *Epilobium* genus, with 21 species naturally distributed in Türkiye (BizimBitkiler 2025; POWO 2025a, 2025b). The *Epilobium* genus is a taxonomically challenging group, attracting the attention of plant taxonomists due to the morphological similarity between species and the high potential for hybridisation. As a result, different views have been proposed regarding classifying the *Epilobium* and *Chamaenerion* genera. Some botanists have considered the *Chamaenerion* genus as a section within *Epilobium*, and this approach has been adopted in various revision works and modern floras. Botanical studies in Türkiye, Iran, South America, Finland, Sweden, and Estonia have used this classification (Güven et al. 2021). The natural distribution area of *E. angustifolium* spans a vast geography from the temperate northern hemisphere to northeastern Mexico and Morocco. *E. angustifolium* , a perennial plant, primarily develops in temperate biomes (BizimBitkiler 2025; POWO 2025a, 2025b).

*E. angustifolium* is distributed in Eurasia and North America, and in Türkiye, it is found in northern and high‐altitude regions. The plant can grow up to 50–250 cm in height, with a stem that is either hairless or slightly hairy. Its leaves are narrow and lance‐shaped, ranging from 2.5 to 13 cm long. The flowers are dark pink or magenta, and the flowering period occurs between June and August. The fruit is a 2.5–8 cm long capsule, with tiny, flat seeds inside. This plant grows in forest edges, meadow areas, and rocky slopes at elevations ranging from 650 to 3050 m. It has been recorded in various regions of Türkiye, particularly in the Black Sea, Central Anatolia, and Eastern Anatolia regions. It is naturally found in places such as Uludağ in Bursa, Kartalkaya in Bolu, Hacıkadın Dere in Ankara, Çambaşı Plateau in Ordu, Sarıkamış in Kars, and Hınıs in Erzurum (Davis 1972; Myerscough 1980).

*E. angustifolium* is known for its anti‐inflammatory, antioxidant, antimicrobial, antiproliferative, and anticancer properties (Schepetkin et al. 2016). The plant treats migraines, insomnia, colds, infections, ulcers, gastritis, dysentery, and intestinal diseases. Its leaves are applied as poultices for burns, bee stings, swelling, and pain, while its infusions are consumed for prostate diseases and urinary tract infections. The plant also exhibits antiseptic and wound‐healing effects on the skin. Studies have shown that *E. angustifolium* contains high amounts of polyphenols and secondary metabolites. It includes various phytochemical compounds such as flavonoids (quercetin, kaempferol, myricetin), phenolic acids (ellagic acid, valoneic acid dilactone), sterols (cholesterol, β‐sitosterol, campesterol, stigmasterol), and tannins. Extracts from *E. angustifolium* have demonstrated antiproliferative effects against cancer cells, particularly by inhibiting the growth of prostate cancer cells. Additionally, its extracts have anti‐inflammatory effects by reducing carrageenan‐induced edema (Kadam et al. 2018).

In recent years, there has been a growing demand for healthier, more natural food products (Kavaz Yüksel et al. 2021). Consumers are increasingly aware of their food's ingredients and actively seek alternatives that provide nutritional benefits while reducing the need for artificial preservatives. This trend has led to significant research into natural food additives, particularly plant‐based extracts, which offer both health benefits and functional properties that can improve food products' quality and shelf life (Petcu et al. 2023).

One area of particular interest is using plant‐derived antioxidants in meat products. Meat, mainly processed meat, is highly susceptible to oxidation, which leads to spoilage, color changes, and the development of undesirable flavors (Manessis et al. 2020). Lipid oxidation is a significant challenge in meat preservation, as it negatively impacts the product's sensory qualities and nutritional value. Traditionally, synthetic antioxidants such as butylated hydroxytoluene and butylated hydroxyanisole have been used to combat oxidation. However, concerns about the potential health risks associated with synthetic additives have fueled the search for natural alternatives (Wang et al. 2021).

One promising natural additive is *E. angustifolium* , commonly known as fireweed (Ferysiuk et al. 2022a). This plant has been recognized for its high phenolic and flavonoid content, contributing to its strong antioxidant properties. *E. angustifolium* has been studied for various medicinal applications, including its anti‐inflammatory, antimicrobial, and antioxidant effects. In food applications, its potential to inhibit oxidation makes it a valuable candidate for use in meat preservation (Nowak et al. 2022).

This study focuses on the application of *E. angustifolium* extract in beef burgers. Researchers prepared four types of beef burgers, incorporating different concentrations of *E. angustifolium* extract as a partial replacement for breadcrumbs, along with a control group that contained no extract. The objective was to evaluate the impact of these extracts on key quality parameters of the burgers, including color stability, pH changes, lipid oxidation, and overall antioxidant activity over an 8‐day refrigerated storage period.

By analyzing these parameters, this research aims to provide insight into the effectiveness of *E. angustifolium* extract as a natural preservative in meat products. If successful, this natural additive could offer a clean‐label solution to improving beef burgers' shelf life and stability, meeting consumer demands for healthier, more natural food options while maintaining product quality.

## 2 Materials and Methods

### 2.1 Collection of Plant Material

The plant materials of *E. angustifolium* were collected on July 16, 2023, from the Çamlık area of Çambaşı Plateau, located at an altitude of 1700 m in the Ordu province. Dr. Alper Durmaz carried out the species identification using the *Flora of Turkey*. The current nomenclature and taxonomic status of *E. angustifolium* were verified through the latest and valid sources. The herbarium material of *E. angustifolium* is registered under accession number OMUB‐5395 at the Herbarium of the Department of Biology, Faculty of Science, Ondokuz Mayıs University.

### 2.2 Plant Material Extraction

The *E. angustifolium* plant was washed with distilled water and dried in the shade for 7 days. Subsequently, the plant samples were dried in an oven at 40°C for 2 days and then ground into a fine powder. 100 g of dried sample was placed into bottles, and 1000 mL of methanol (1:10 ratio g/mL) was added. The solution was stirred occasionally and kept in the dark for 72 h. The solutions were then filtered through Watmann filter paper and evaporated using a rotary evaporator (Heidolph, Germany) at 40°C. The solid extracts were stored at 4°C and prepared for further use (Aytar and Aydın 2024).

1 g of dried powdered *E. angustifolium* was separately mixed with 10 mL of methanol. First, the mixtures underwent ultrasonic treatment for 30 min. After this step, the samples were placed in a shaker and incubated at room temperature in the dark for 24 h to enhance the release of bioactive compounds. Once the incubation was complete, the extracts were filtered through standard filter paper to remove large particles. Then, a second filtration was performed using a 0.45 μm syringe filter to eliminate smaller particles. This extraction process, which includes ultrasonication, prolonged incubation, and two‐step filtration, was designed to ensure maximum transfer of the target compounds from the dried powder into the extraction solution. The final clear filtrates were ready for analysis (Akbulut et al. 2021; Uysal et al. 2016).

### 2.3 Determination of Total Phenolic Content, Total Flavonoid Content, and Antioxidant Activity of *Epilobium angustifolium* Extract

The free radical scavenging activities of extracts were determined using DPPH (1,1‐diphenyl‐2‐picrylhydrazyl) according to Lee, Hendricks, et al. (1998) and Lee, Mbwambo, et al. (1998). The absorbance was recorded at 517 nm using a spectrophotometer (UV‐160 A, UV–Visible Recording Spectrophotometer, Shi‐madzu, Tokyo, Japan). The results were expressed as a percentage of free radical scavenging activity (%).

The total phenolic contents of extracts were determined using the Folin–Ciocalteu method described by Yoo et al. (2004). The absorption was measured at 750 nm against a reagent blank in a UV–vis spectrophotometer. The results are provided as mg gallic acid equivalents (GAE)/100 mL.

The total flavonoid contents of extracts were determined using the method reported by Chen and Chen (2011). The absorbance of the mixture was measured at 510 nm. The catechin was used as a standard, and the results were expressed as mg of catechin equivalents (mg ce/100 mL).

A radical reagent was prepared by mixing 100 mL of sodium acetate buffer (300 mmol/L, pH 3.6), 10 mL of 2,4,6‐tri(2‐pyridyl)‐s‐triazine solution (10 mmol/L in 40 mmol/L hydrochloric acid solution) and 10 mL of ferric chloride hexahydrate solution (20 mmol/L) for FRAP analysis. Then, 75 μL of the sample, 2.25 mL of radical reagent, and 225 μL of distilled water were mixed and incubated at room temperature for 30 min. The absorbance of samples was recorded at 593 nm. Additionally, the standard curve for FRAP was prepared by iron (II) sulfate heptahydrate, and results were expressed as mg/kg on a dry basis (Aktas and Tontul 2021).

### 2.4 Gas Chromatography–Mass Spectrometry Analysis of Fatty Acids

5 g of the plant material was finely ground to determine the fatty acid composition using a laboratory mill. The powdered sample was enclosed in standard filter paper and subjected to Soxhlet extraction in an automated Soxhlet apparatus. Hexane was used as the extraction solvent, and the process was carried out for 4 h. The solvent was evaporated upon completion, and the extracted oil was transferred to vials for further analysis.

For the preparation of fatty acid methyl esters (FAMEs), approximately 0.1 g of the extracted oil was placed in a 5 mL glass tube. Subsequently, 2 mL of n‐hexane was added, and the mixture was thoroughly vortexed. Then, 0.2 mL of 2 N methanolic potassium hydroxide (KOH) was introduced, and the tube was tightly sealed and vigorously shaken for 30 s. The mixture was centrifuged to promote phase separation and left to stand briefly. The upper phase, containing the methyl esters, was carefully collected using a Pasteur pipette and transferred into vials for GC–MS analysis.

GC–MS analysis was performed using an Agilent gas chromatograph (Agilent Technologies, Santa Clara, CA, USA) equipped with an HP‐88 capillary column (60 m × 0.25 mm × 0.20 μm). High‐purity helium (> 99.99%) was employed as the carrier gas at a constant flow rate of 1.0 mL/min. The initial oven temperature was set at 140°C and maintained for 5 min, followed by a gradual increase at a rate of 4°C/min until reaching 250°C, where it was held constant for 10 min. The split injection mode was applied, with an injection volume of 1 μL and a split ratio of 1:50. Mass spectrometric detection was performed in electron ionization (EI) mode at an ionization energy of 70 eV. The ion source temperature was maintained at 230°C, and mass scanning was conducted over an *m*/*z* range of 30–550 (Kumar et al. 2018; Zhang et al. 2022).

### 2.5 Extraction and Quantification of Phenolic Compounds via High‐Performance Liquid Chromatography With Diode‐Array Detection (HPLC‐DAD)

To extract phenolic compounds, 1 g of finely powdered plant material was mixed with 10 mL of methanol. Ultrasonic treatment was applied for 30 min to enhance the release of phenolic constituents into the solvent. The mixture was subsequently incubated at room temperature in the dark with continuous agitation for 24 h to maximize extraction efficiency. Following incubation, the extract was filtered through standard filter paper to remove coarse particles. A subsequent filtration step was performed using a 0.45 μm syringe filter to eliminate residual fine particulates, yielding a clarified extract for chromatographic analysis.

Chromatographic separation was performed using an ACE 5 C18 column (250 mm × 4.6 mm, 5 μm particle size). The mobile phase consisted of acetonitrile (Solvent A) and an aqueous solution of 1.5% acetic acid (Solvent B). A gradient elution program was employed, starting with 15% Solvent A and 85% Solvent B, gradually increasing to 40% Solvent A and 60% Solvent B over 29 min. The HPLC system was equipped with a 1260 DAD WR detector, monitoring absorbance at 250, 270, and 320 nm. A 1260 Quaternary Pump maintained a constant flow rate of 0.7 mL/min, while a 1260 Vialsampler injected 10 μL of the prepared extract into the system. The column temperature was precisely regulated at 35°C using a G7116A column oven.

Phenolic constituents were quantified using calibration curves generated from six standard solutions at concentrations of 25, 50, 75, 100, 200, and 300 μg/mL. Integrating ultrasonic‐assisted extraction, extended incubation, sequential filtration, and high‐performance liquid chromatography (HPLC) enabled efficient recovery and precise quantification of phenolic compounds (Alan 2023; Seal 2016).

### 2.6 Preparation of Plant Samples and Standard Solutions for ICP‐MS Analysis

For ICP‐MS analysis, plant samples were processed in dried and finely ground form. A precisely measured quantity of plant material (ranging from 0.30 to 0.80 g, with an accuracy of ±0.0001 g) was placed into Teflon digestion vessels. Subsequently, 6–10 mL of concentrated ultrapure nitric acid (67%–68%) and up to 1 mL of ultrapure water were added. The vessels were securely sealed, and sample digestion was carried out using a microwave digestion system under a 40‐min programmed cycle.

Following digestion, the resulting solutions were passed through a 0.45 μm syringe filter to ensure clarity. The filtered extracts were then transferred into 50 mL volumetric flasks and diluted to volume with a solution containing 2% nitric acid in ultrapure water.

Metal ion standards (Mg, K, Ca, C, and H) were prepared from stock solutions at an initial 1000 mg/L concentration. Working solutions, covering a concentration range of 0–500 μg/L, were obtained by serial dilution and adjusted to a final volume of 50 mL. Each sample was analyzed in triplicate using an ICP‐MS system, and a calibration curve was generated. The calibration model was optimized to maintain an *R* ^2^ value between 0.99 and 1.00, ensuring reliable measurement accuracy (Filipiak‐Szok et al. 2015).

### 2.7 Molecular Docking Analysis

In this study, molecular docking simulations were carried out to examine the interactions between the major compounds identified in GC–MS and LC–MS analyses and a specific target protein, 5EK8, the crystal structure of a 9R‐lipoxygenase from Cyanothece PCC8801, at a resolution of 2.7 Å. In the first step, related to the preparation of the PDB file, the protein crystal structure (5EK8) is downloaded from the PDB (www.rscb.com) (Noshad et al. 2023b). Before the docking process, all water molecules and cofactors were removed to prevent interference, and polar hydrogen atoms were incorporated using AutoDockTools (ADT) to optimize the protein structure for docking studies. The ligand structures were obtained from the PubChem database in SDF format and subsequently converted to PDB format using Discovery Studio Visualizer. The binding site was defined using AutoGrid, with the grid centred on the active site and set to dimensions of 40 points in each direction, with a grid spacing of 0.375 Å. Docking simulations were performed using AutoDock Vina, generating 10 possible binding poses for each ligand to assess their binding affinity. The energy range was fixed at 9 kcal/mol, and the exhaustiveness parameter was set to 1000 to enhance the reliability of the docking results. The docking outcomes were evaluated based on binding energy, ligand efficiency (LE), fit quality (FQ), *p*IC_50_, and the estimated inhibition constant (Ki). The binding poses and molecular interactions were visualized in both 2D and 3D formats using BIOVIA Discovery Studio Visualizer (Biovia and Systèmes 2016), facilitating a detailed examination of ligand‐protein interactions. This study offers critical insights into these compounds' binding potential and pharmacological significance concerning the selected target protein.

### 2.8 Preparation of Beef Burgers

Four types of beef burgers were prepared depending on the addition of increasing doses of extracts from *E. angustifolium* (fireweed), 1, 3, and 9 g substituted by breadcrumbs, including a control group (no added extract). In the basic formulation, the beef burger groups' ingredients are shown in Table 1.

**TABLE 1:** Formulating beef burgers.

Ingredients (g)	Treatments			
	C	T1	T2	T3
Meat	1000	1000	1000	1000
Breadcrumbs	120	119	117	111
Distilled water	70	70	70	70
Salt	10	10	10	10
*Epilobium angustifolium* (fireweed) extract (ppm)	—	100	300	900

*Note:* T1: 1 g *Epilobium angustifolium* extract added beef burger; T2; 3 g *Epilobium angustifolium* (fireweed) extract added beef burger; T3; 9 g *Epilobium angustifolium* extract added beef burger.

Abbreviation: C, Control.

Fresh bovine *Longissimus thoracis et lumborum* muscle (24 h post‐mortem) obtained from a butcher in Konya, after being separated from the connective tissue, was first minced twice in a meat grinder (Kitchen Aid, Classic Model, USA) with a plate with 3 mm diameter holes and divided into four groups. The other ingredients were added and mixed for about 4 min. Each beef burger (approximately 40 ± 0.1 g each) was shaped by hand into rounds (35 mm in diameter and 10 mm thick) and placed individually on styrofoam trays. The burgers were then wrapped with polyvinyl chloride film and kept at 4°C ± 1°C for 8 days. A total of 72 beef burgers were produced: three samples per treatment × four treatments (C, T1, T2 and T3) × three storage periods (1, 4 and 8 days) × two independent replicates (using similar production processes) (Babaoğlu et al. 2022).

Chilled beef burger samples were analyzed for instrumental color, pH, and thiobarbituric acid‐reactive substances (TBARS) at sampling times (Days 1, 4 and 8).

### 2.9 pH Measurements

The pH values of the beef burgers were determined using a pH meter and measuring at three different locations of each sample. The pH meter used for this purpose was calibrated before the analysis using buffer solutions with pH 4, 7, and 10 (Lambooij et al. 1999).

### 2.10 Color Measurements

L* (lightness), a* (redness), and b* (yellowness) parameters of the samples were determined using a colourimeter (Konica, Minolta CR 400, Osaka, Japan) with illuminant D65, 2° observer angle, diffuse/O mode, 8 mm aperture for illumination and 8 mm for measurement. Before each measuring session, the chromometer was calibrated on the CIE color space system using a white tile. The L* value indicates lightness (L* = 0 darkness, L* = 100 lightness); the a* value indicates redness (+60 = red, −60 = green); and the b* value indicates yellowness (+60 = yellow, −60 = blue). Color measurement was made perpendicular to the sample surface at four randomly different locations per sample at room temperature (≈22°C), and mean values (L*, a*, and b*) from each sample were analyzed (Babaoğlu et al. 2022).

### 2.11 Thiobarbituric Acid‐Reactive Substances (TBARS)

To calculate the TBARS value, an indicator of lipid oxidation, a spectrophotometric measurement (UV‐160 A, UV–Visible Recorder Spectrophotometer, Shimadzu, Tokyo, Japan) was performed at 530 nm. Then, the absorption values obtained were multiplied by the coefficient 7.03, and the TBARS number was calculated as mg malonaldehyde/kg sample (Tarladgis et al. 1960).

### 2.12 Statistical Analysis

Four treatments (C, T1, T2, and T3) and three storage durations (1, 4, and 8 days) were used in a completely randomized factorial design with two independent replications. The generalized linear mixed model was used to perform an analysis of variance (ANOVA) for the statistical analysis of the pH, TBARS, and color values. The replication was regarded as a random element, whereas the plant extract treatment, storage duration, and interaction were examined as fixed factors. To ascertain the differences between the means at a 5% significant level, Tukey Multiple Comparison Tests were employed in conjunction with the plant extract treatment, storage period, and the interaction between the plant extract treatment and storage time.

## 3 Results

### 3.1 Total Phenolic Content and Antioxidant Properties of *Epilobium angustifolium* Extract

Table 2 indicates the contents of antioxidant activity (DPPH and FRAP), total phenolic (TPC), and total flavonoid (TFC) of *Epilobium angustifolium* (fireweed) extract. The DPPH, FRAP, TPC, and TFC were found to be 48.80% ± 3.74%, 2198.05 ± 78.56 mg/L, 1263.48 ± 12.13 mg GAE/L, and 278.43 ± 3.27 mg ce/L, respectively.

**TABLE 2:** The antioxidant activity (DPPH and FRAP), total phenolic (TPC) and total flavonoid (TFC) contents of *Epilobium angustifolium* extract.

Analysis	*Epilobium angustifolium* extract
DPPH (%)	48.80 ± 3.74
FRAP (mg/L)	2198.05 ± 78.56
TFC (mg ce/100 mL)	278.43 ± 3.27
TPC (mg GAE/100 mL)	1263.48 ± 12.13

### 3.2 GC–MS Analysis and Phytochemical Profile

The GC–MS analysis of the sample identified a diverse range of phytochemical compounds characterized by varying retention times and retention indices (Table 3). The major constituents included α‐Pinene (22.86%) and β‐Thujone (18.43%), which exhibited the highest abundance among the detected compounds. Other significant components were Furan, 2‐pentyl‐ (7.60%), Hexanal (6.32%), and D‐Limonene (5.72%), reflecting the complexity of the volatile profile. Additionally, Trans‐2‐Methylcyclopentanol (5.54%), Tert‐Butylcyclohexane (5.06%), and p‐Cymene (4.99%) contributed to the overall chemical diversity.

**TABLE 3:** Phytochemical compounds of *Epilobium angustifolium* methanol extracts.

No	Retantion time (min)	Retantion index	Name of the compund	Content %
1	4.280	709	Trans‐2‐metilsiklopentanol	5.54
2	6.176	802	Hexanal	6.32
3	6.298	806	Tert‐butylcyclohexane	5.06
4	11.181	943	α‐Pinen	22.86
5	12.408	972	Benzaldehyde	2.79
6	13.214	991	beta.‐Pinene	2.81
7	13.955	1008	Furan, 2‐pentyl‐	7.60
8	15.642	1047	p‐Cymene	4.99
9	15.851	1052	D‐Limonene	5.72
10	20.243	1144	5‐Methyl‐1,4‐hexadiene	5.92
11	20.573	1151	β‐Thujone	18.43
12	40.818	1611	trans‐Calamenene	3.41

The retention times of the identified compounds ranged from 4.280 to 40.818 min, with retention indices varying between 709 and 1611, indicating the presence of both low and high molecular weight compounds. The sample primarily contained monoterpenes, oxygenated terpenes, and aromatic hydrocarbons, suggesting a complex composition influenced by structural variations and volatility differences. Notably, Benzaldehyde (2.79%), β‐Pinene (2.81%), and trans‐Calamenene (3.41%) were detected in lower concentrations, but their presence further supported the chemical complexity of the sample.

The results demonstrate that the sample comprises a broad spectrum of volatile organic compounds, contributing to its unique phytochemical profile. These constituents' relative abundance and diversity suggest potential variations in physicochemical properties, which may influence further analytical and functional studies.

### 3.3 Quantification of Phenolic and Flavonoid Compounds via HPLC Analysis

The analysis revealed the presence of various phenolic and flavonoid compounds in the sample (Table 4). Ascorbic acid (7254.61 mg/L) was identified as the most abundant compound, highlighting its significant antioxidant capacity and biological role. Gallic acid (5147.10 mg/L) was the second most prevalent compound, known for its strong antioxidant and antimicrobial properties. Among the phenolic acids, 3,4‐dihydroxybenzoic acid (193.49 mg/L) was present at a notable level, while vanillic acid (4.04 mg/L) and chlorogenic acid (14.70 mg/L) were detected in relatively lower concentrations.

**TABLE 4:** HPLC results of *Epilobium angustifolium* methanol extracts.

No	Compounds	Content (mg/L)
1	Ascorbic acid	7254.61
2	Gallic acid	5147.10
3	3,4 hydroxy benzoic acid	193.49
4	Vanillic acid	4.04
5	Coumaric acid	30.43
6	Rosmarinic acid	102.07
7	Progallol	1175.63
8	Chloragenic acid	14.70
9	Oleuropein	36.60
10	Catechin	416.60
11	Epicatechin	1085.81
12	Rutin	11.57
13	Quercetin	42.70
14	Baicalin	2.37

Rosmarinic acid (102.07 mg/L) and p‐coumaric acid (30.43 mg/L) were found at moderate levels among the phenolic compounds, both recognized for their anti‐inflammatory and antioxidant properties. Progallol (1175.63 mg/L) and epicatechin (1085.81 mg/L) were detected in relatively high concentrations, suggesting a strong redox activity within the sample. Among flavonoids, catechin (416.60 mg/L) and rutin (11.57 mg/L) were identified, whereas quercetin (42.70 mg/L) and oleuropein (36.60 mg/L) were found at comparatively lower levels. Baicalin (2.37 mg/L) was recorded as the least abundant compound.

Overall, the sample exhibited a high ascorbic and gallic acid concentration, with a diverse distribution of phenolic acids and flavonoids. Given the wide range of biological activities associated with these compounds, the obtained data provide valuable insights into the pharmacological and functional potential of the sample.

### 3.4 Macro Elemental Composition and Elemental Analysis of *E. angustifolium*

The macro elemental composition and elemental analysis of *E. angustifolium* were determined to assess its nutritional and chemical characteristics. The results indicate that *E. angustifolium* contains significant levels of essential macroelements (Table 5). Magnesium (Mg) was measured at 2705.68 ± 28.50 mg per 100 g, while potassium (K) was found at 6293.27 ± 43.07 mg per 100 g. Calcium (Ca) was present at the highest concentration, reaching 10,000.22 ± 22.04 mg per 100 g, highlighting the plant's potential as a calcium‐rich source.

**TABLE 5:** Macro elemental composition and elemental analysis of *Epilobium angustifolium* methanol extracts.

No	Plant species	Macro elements mg (100 g)^−1^			Elemental analysis (%)	
		Mg	K	Ca	Carbon	Hydrogen
1	*Epilobium angustifolium*	2705.68 ± 28.50	6293.27 ± 43.07	10000.22 ± 22.04	44.09	5.53

In addition to macroelement analysis, the elemental composition of carbon and hydrogen content was examined. The carbon content was determined as 44.09%, whereas the hydrogen content was measured at 5.53%. These values provide insight into the biochemical structure and potential applications of *E. angustifolium* in various nutritional and industrial contexts. The data suggest that this species could be a valuable source of essential minerals and organic components, supporting its relevance in dietary and pharmaceutical studies.

### 3.5 Result of Molecular Docking

The molecular docking analysis results indicate that the investigated compounds exhibit varying binding affinities with the target protein 5EK8. Gallic acid demonstrated the strongest binding affinity among the analyzed compounds, with a binding energy of −6.8 kcal/mol. It exhibited a LE of 0.378 and a FQ of 0.598, suggesting a favorable binding profile. The estimated inhibition constant (Ki) was calculated as 10.365 μM, and its *p*IC_50_ value was determined to be 4.857, indicating a strong inhibitory potential against the target protein.

**FIGURE 2:**
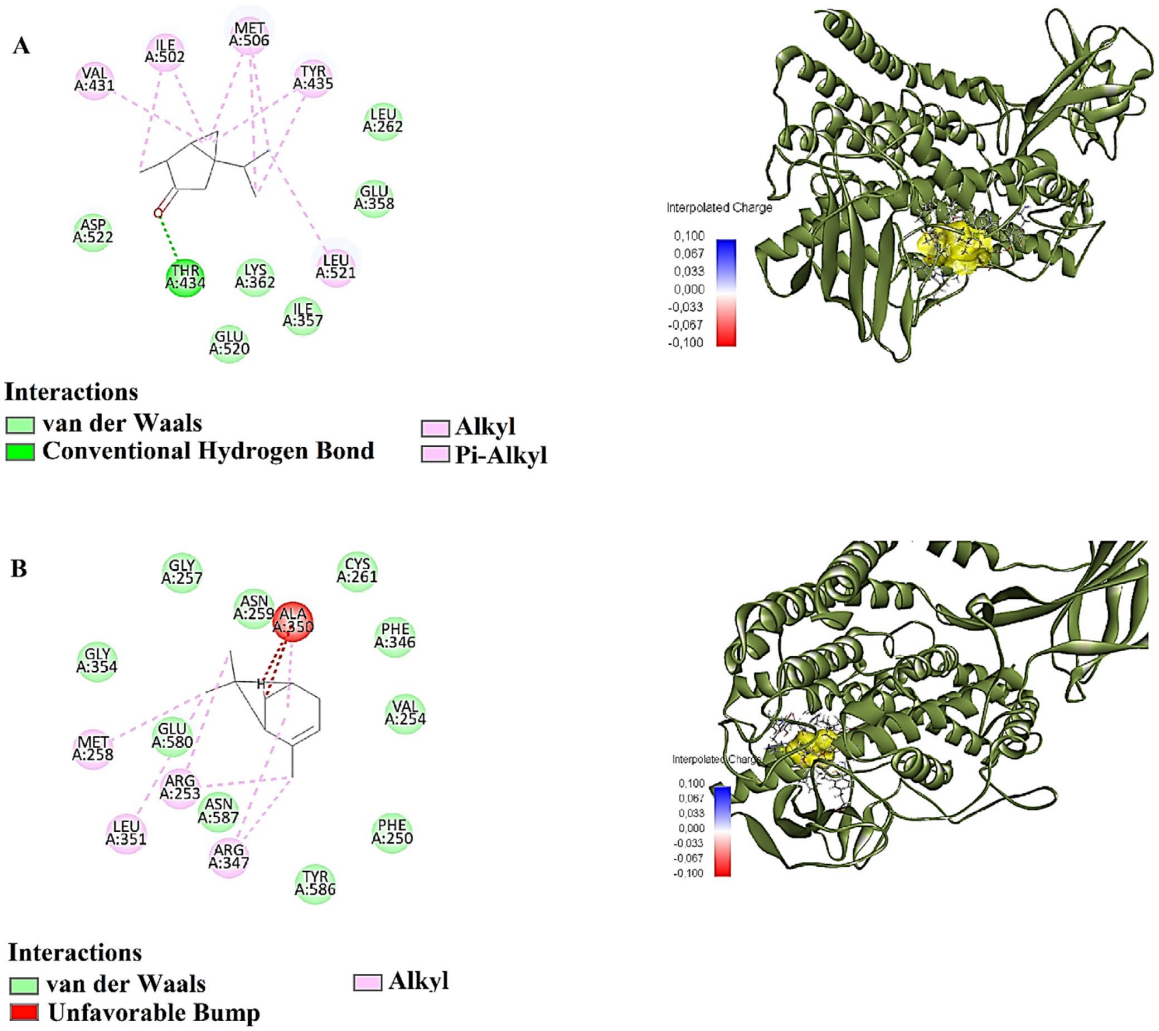
2D and 3D Interactions of (A) beta‐THUJONE (B) alpha‐PINENE with 9R‐lipoxygenase (5EK8).

Beta‐thujone exhibited the second‐highest binding affinity, with a binding energy of −6.3 kcal/mol. It displayed a LE of 0.233 and an FQ value of 0.577, indicating moderate binding efficiency. The estimated Ki was 24.103 μM, with a *p*IC_50_ value of 4.500, suggesting a moderate inhibitory effect on the target protein. Similarly, alpha‐pinene exhibited a binding energy of −5.8 kcal/mol, a LE of 0.223, and an FQ value of 0.531 (Table 6; Figure 2). The estimated Ki was 56.049 μM, with a *p*IC_50_ value of 4.143, indicating a weaker binding affinity than beta‐thujone and gallic acid.

**TABLE 6:** Results of binding interactions of the compounds with target 5EK8.

	Binding energy (kcal/mol)	Ligand efficiency	Fit quality (FQ)	Estimated inhibition constant (Ki) (μM)	*p*IC_50_
Beta‐THUJONE	−6.3	0.233	0.577	24.103	4.500
Alpha‐PINENE	−5.8	0.223	0.531	56.049	4.143
Ascorbic acid	−5.1	0.255	0.457	182.672	3.643
Gallic acid	−6.8	0.378	0.598	10.365	4.857

**FIGURE 3:**
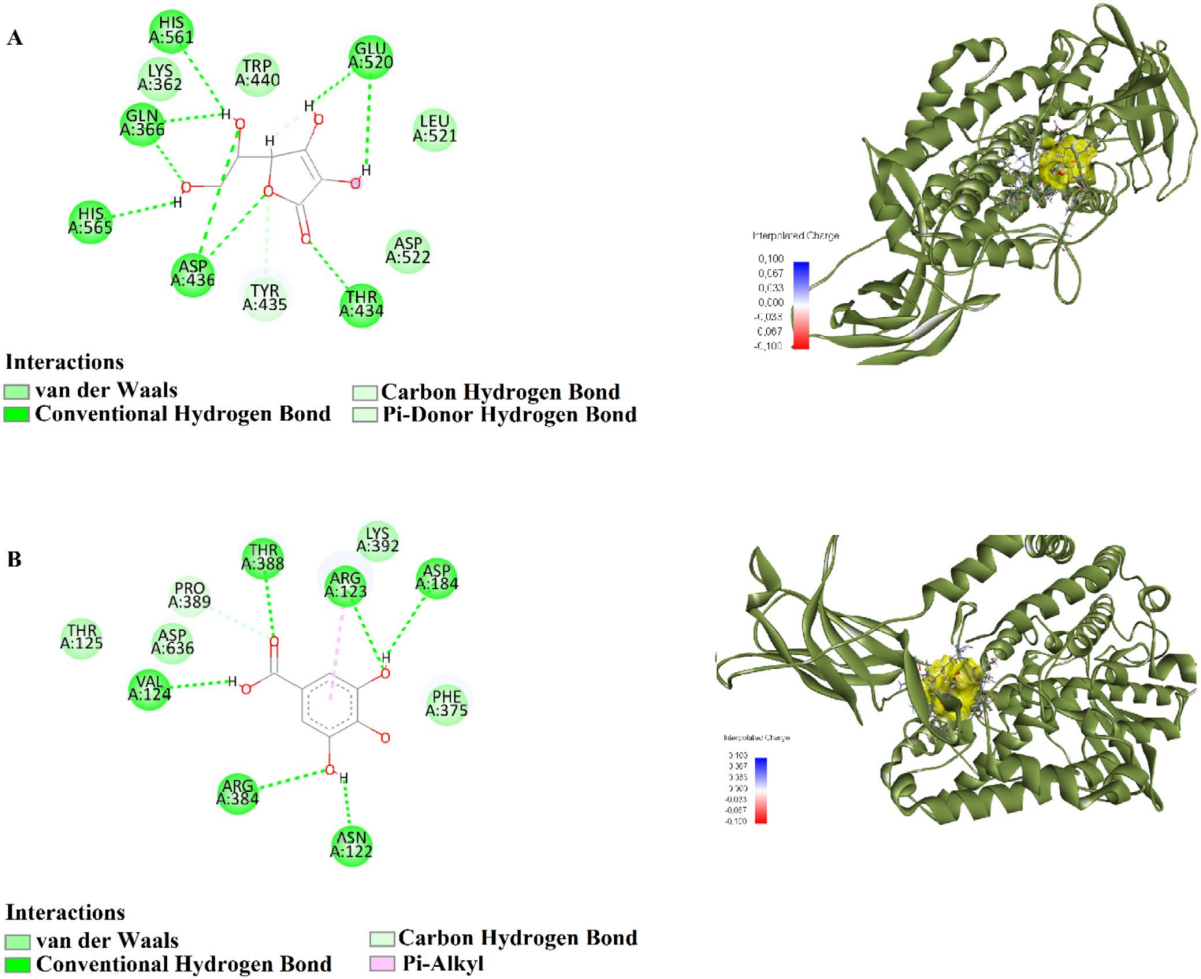
2D and 3D Interactions of (A) ascorbic acid (B) gallic acid with 9R‐lipoxygenase (5EK8).

Conversely, ascorbic acid exhibited the lowest binding affinity, with a binding energy of −5.1 kcal/mol (Table 6; Figure 3). It displayed a LE of 0.255 and an FQ value of 0.457, suggesting relatively low binding efficiency. The estimated Ki was 182.672 μM, while the *p*IC_50_ value was calculated as 3.643, indicating a weak inhibitory interaction with the 5EK8 target protein.

Overall, the results suggest that gallic acid exhibits the strongest binding affinity and inhibitory potential against the 5EK8 target protein, followed by beta‐thujone and alpha‐pinene, which show moderate binding interactions. In contrast, ascorbic acid displayed the lowest binding strength.

Molecular docking analysis revealed the interactions of the investigated compounds with the 5EK8 target protein through various amino acid residues (Table 7). Beta‐thujone formed a conventional hydrogen bond with THR434 at a distance of 2.34 Å. Additionally, it exhibited alkyl and pi‐alkyl interactions with several amino acid residues, including VAL431, MET506, LEU521, ILE502, and TYR435. This binding profile suggests that beta‐thujone primarily interacts with the protein surface through hydrophobic interactions, contributing to its overall stability.

**TABLE 7:** Docking of predicted interactions of docked conformations against 5EK8.

Ligand	Amino acids	Interacting	Distance
Beta‐THUJONE	A:THR434:HG1 ‐: [001:O1	Conventional hydrogen bond	2.34
	A:VAL431 ‐: [001	Alkyl	5.19
	:[001:C1 ‐ A:MET506	Alkyl	4.19
	:[001:C3 ‐ A:MET506	Alkyl	4.18
	:[001:C3 ‐ A:LEU521	Alkyl	4.95
	:[001 ‐ A:ILE502	Alkyl	4.43
	:[001 ‐ A:MET506	Alkyl	4.65
	:[001:C10 ‐ A:ILE502	Alkyl	3.37
	A:TYR435 ‐: [001:C1	Pi‐alkyl	5.32
	A:TYR435 ‐: [001	Pi‐alkyl	5.47
Alpha‐PINENE	A:ARG347 ‐: [001	Alkyl	5.27
	A:ALA350 ‐: [001	Alkyl	3.56
	:[001:C7 ‐ A:ARG253	Alkyl	4.99
	:[001:C8 ‐ A:MET258	Alkyl	3.84
	:[001:C8 ‐ A:LEU351	Alkyl	4.80
	:[001:C10 ‐ A:ARG253	Alkyl	3.90
	:[001:C10 ‐ A:ARG347	Alkyl	3.77
Ascorbic acid	A:GLN366:HE22 ‐: [001:O4	Conventional hydrogen bond	1.57
	A:THR434:HG1 ‐: [001:O2	Conventional hydrogen bond	2.21
	A:ASP436:HN ‐: [001:O1	Conventional hydrogen bond	2.52
	A:ASP436:HN ‐: [001:O3	Conventional hydrogen bond	2.79
	:[001:H1 ‐ A:GLN366:OE1	Conventional hydrogen bond	1.77
	:[001:H1 ‐ A:HIS561:NE2	Conventional hydrogen bond	2.67
	:[001:H2 ‐ A:HIS565:NE2	Conventional hydrogen bond	2.25
	:[001:H7 ‐ A:GLU520:O	Conventional hydrogen bond	2.05
	:[001:H8 ‐ A:GLU520:O	Conventional hydrogen bond	2.86
	A:TYR435:HA ‐: [001:O1	Carbon hydrogen bond	2.22
	:[001:H4 ‐: [001:O5	Carbon hydrogen bond	2.01
	:[001:H6 ‐ A:GLU520:O	Carbon hydrogen bond	2.76
	:[001:H2 ‐ A:HIS565	Pi‐donor hydrogen bond	2.61
Gallic acid	A:ARG123:HE ‐: [001:O1	Conventional hydrogen bond	2.75
	A:ARG384:HE ‐: [001:O3	Conventional hydrogen bond	2.36
	A:THR388:HG1 ‐: [001:O5	Conventional hydrogen bond	2.43
	:[001:H1 ‐ A:ASP184:OD1	Conventional hydrogen bond	2.69
	:[001:H3 ‐ A:ASN122:O	Conventional hydrogen bond	1.63
	:[001:H5 ‐ A:VAL124:O	Conventional hydrogen bond	2.27
	A:THR388:HB ‐: [001:O5	Carbon hydrogen bond	3.00
	A:PRO389:HD1 ‐: [001:O5	Carbon hydrogen bond	2.53
	:[001 ‐ A:ARG123	Pi‐alkyl	3.42

Alpha‐pinene established alkyl interactions with ARG347, ALA350, ARG253, MET258, and LEU351. The observed binding distances ranged from 3.56 to 5.27 Å, indicating the predominance of hydrophobic interactions. This interaction pattern suggests that the binding stability of alpha‐pinene with the target protein may be lower than that of beta‐thujone, as it lacks strong polar interactions.

Ascorbic acid exhibited the highest number of interactions via conventional hydrogen bonding, one of the strongest binding types. It formed hydrogen bonds with GLN366, THR434, ASP436, HIS561, HIS565, and GLU520, with distances ranging from 1.57 to 2.86 Å. Carbon‐hydrogen bonds with TYR435 and pi‐donor hydrogen bonds with HIS565 were also detected. These findings indicate that ascorbic acid primarily binds through hydrophilic interactions, forming multiple hydrogen bonds that enhance its binding strength and stability with the target protein.

Gallic acid demonstrated strong binding interactions with the target protein, forming hydrogen bonds with ARG123, ARG384, THR388, ASP184, and ASN122 at distances of 1.63 to 2.75 Å. Furthermore, it established an additional hydrogen bond with VAL124 and carbon‐hydrogen bonds with THR388 and PRO389. A pi‐alkyl interaction at 3.42 Å with ARG123 suggests that the aromatic characteristics of gallic acid contribute to its binding stability.

### 3.6 pH Values of Beef Burgers

Table 8 presents the pH values of beef burgers during the storage period. On the first day, the treatment with plant extract did not significantly affect the pH values (*p* > 0.05), with pH values ranging from 5.10 to 5.64.

**TABLE 8:** pH values and color properties of beef burgers.

Analysis	Treatments	Storage period (day)		
		1	4	8
pH	C	5.62 ± 0.01^aA^	5.29 ± 0.02^aB^	5.17 ± 0.01^aC^
	T1	5.10 ± 0.58^aA^	5.27 ± 0.01^aA^	5.10 ± 0.04^abA^
	T2	5.51 ± 0.00^aA^	5.16 ± 0.02^bB^	5.06 ± 0.01^bcC^
	T3	5.64 ± 0.01^aA^	5.24 ± 0.03^abB^	5.00 ± 0.01^cC^
L*	C	55.56 ± 0.22^aAB^	52.92 ± 1.53^aB^	56.85 ± 0.50^aA^
	T1	51.35 ± 1.00^bcA^	48.45 ± 0.28^bB^	51.67 ± 0.16^cA^
	T2	48.78 ± 1.50^cB^	50.05 ± 0.02^abAB^	53.60 ± 0.05^bA^
	T3	53.46 ± 0.23^abB^	51.92 ± 0.34^aC^	56.13 ± 0.08^aA^
a*	C	13.96 ± 0.45^bA^	14.21 ± 0.85^aA^	15.46 ± 0.64^cA^
	T1	15.59 ± 0.32^aB^	14.09 ± 0.70^aB^	18.73 ± 0.06^aA^
	T2	13.41 ± 0.01^bB^	12.45 ± 0.72^aB^	17.05 ± 0.16^bA^
	T3	9.49 ± 0.33^cA^	12.00 ± 1.53^aA^	12.75 ± 0.06^dA^
b*	C	17.82 ± 0.30^bA^	16.33 ± 1.26^abA^	16.38 ± 0.16^bA^
	T1	15.41 ± 0.83^cAB^	14.47 ± 0.05^bB^	16.85 ± 0.07^bA^
	T2	16.53 ± 0.42^bcA^	15.67 ± 0.01^abA^	17.03 ± 1.24^bA^
	T3	20.68 ± 0.20^aA^	18.88 ± 1.03^aA^	21.67 ± 0.60^aA^

*Note:* Mean ± std. dev. Within the same row, values with different uppercase superscript letters indicate significant differences (*p* < 0.05) Within the same column, values with different lowercase superscript letters indicate significant differences (*p* < 0.05). T1: 1 g *Epilobium angustifolium* (fireweed) extract added beef burger; T2: 3 g *Epilobium angustifolium* extract added beef burger; T3: 9 g *Epilobium angustifolium* extract added beef burger.

Abbreviation: C, Control.

However, significant effects of the treatment were observed on Day 4 (for only T2) and Day 8 (for T2 and T3 only) (*p* < 0.05). These days, the treated samples' pH values were lower compared to the control group. When analyzing the pH values over the entire storage period, it was noted that the pH decreased in all groups, except for T1, compared to the first day. Specifically, the pH in the control group decreased from 5.62 to 5.17, from 5.51 to 5.06 in T2, and from 5.64 to 5.00 in T3.

### 3.7 Color Properties of Beef Burgers

The plant extract obtained from *E. angustifolium* treatment effects on color changes of beef burgers during refrigerated storage is shown in Table 8.

According to the results of the present study, the use of plant extract obtained from *E. angustifolium* resulted in the treated groups (except T3) showing darker (*p* < 0.05) compared to the control group on the first day of the study (Table 3). On Days 4 and 8, the L* value was significantly affected (*p* < 0.05) in the groups containing 1 and 3 g extracts, while the addition of the highest concentration (9 g) yielded results like the control group (*p* > 0.05). During storage, the L* value increased in the T2 group, while fluctuations were observed in the control, T1, and T3 groups (*p* < 0.05).

The use of plant extract obtained from *E. angustifolium* in the formulation caused similar effects (*p* > 0.05) on the b* value of beef burger samples on the 1st and 4th days of the study. When 1 g extract was used in the formulation, less yellowness was observed, while the addition of 9 g extract resulted in more yellowness compared to the control group samples (*p* < 0.05). On the last day of the study, a higher b* value was observed in the group with 9 g plant extract, leading to a discolouration compared to the control group. Throughout the storage process, no significant change (*p* > 0.05) in the b* value was observed in the control, T2, and T3 groups, while the b* value of the T1 group was measured as the lowest on Day 4 and the highest on Day 8 (*p* < 0.05).

When the redness (a*) values of the samples were examined, no significant difference was observed between the groups on the 4th day of the study (*p* > 0.05). However, on the 1st and 8th days, it was determined that the samples containing 9 g plant extract had less redness than the control group (*p* < 0.05). On the first day of the study, the T2 and control groups had similar a* values, while samples with 1 g extract exhibited more redness (*p* < 0.05). By the end of the study, samples containing 3 g plant extract also displayed more redness than the control group (*p* < 0.05), like the 1 g group (*p* > 0.05). After 8 days of storage, the a* value of the T3 group samples decreased compared to the control group, while the samples containing 1 and 3 g extract had higher redness values on the final day of the study (*p* < 0.05).

### 3.8 TBARS Numbers of Beef Burgers

Table 9 shows the TBARS numbers of beef burgers elaborated with *E. angustifolium* (fireweed) extract throughout 8 days of refrigerated storage. TBARS numbers ranged between 0.197 and 0.830 mg MA/kg samples in the initial storage period. Regarding the evolution of oxidative reactions, all burgers showed an increasing trend over time wherein values in the period 0.710–0.931 mg MA/kg sample were observed after 8 days. However, as illustrated in Table 4, the T3 group displayed higher TBARS values than other treatments, showing that adding 9 g of fireweed results in an oxidant effect during the storage period, except on day 8. This could be partly explained by these extracts possessing prooxidant and antioxidant activities under certain circumstances.

**TABLE 9:** The TBARS number (mg MA/kg sample) of beef burgers.

Analysis	Treatments	Storage period (day)		
		1	4	8
TBARS number (mg MA/kg sample)	C	0.641 ± 0.002^bC^	0.687 ± 0.012^bB^	0.931 ± 0.006^aA^
	T1	0.197 ± 0.000^dC^	0.578 ± 0.003^cB^	0.752 ± 0.010^cA^
	T2	0.278 ± 0.005^cC^	0.591 ± 0.009^cB^	0.710 ± 0.010^dA^
	T3	0.830 ± 0.020^aA^	0.782 ± 0.002^aA^	0.791 ± 0.005^bA^

*Note:* Mean ± std. dev. Within the same row, values with different uppercase superscript letters indicate significant differences (*p* < 0.05) Within the same column, values with different lowercase superscript letters indicate significant differences (*p* < 0.05). T1: 1 g *Epilobium angustifolium* extract added beef burger; T2; 3 g *Epilobium angustifolium* extract added beef burger; T3; 9 g *Epilobium angustifolium* extract added beef burger.

Abbreviation: C, Control.

## 4 Discussion

### 4.1 Total Phenolic Content and Antioxidant Properties of *Epilobium angustifolium* Extract

*E. angustifolium* exhibited DPPH, FRAP, TPC, and TFC values of 48.80% ± 3.74%, 2198.05 ± 78.56 mg/L, 1263.48 ± 12.13 mg GAE/L, and 278.43 ± 3.27 mg ce/L, respectively. The ability of *E. angustifolium* to inhibit DPPH radicals at potent levels has already been reported by Shikov et al. (2006) and Wojdyło et al. (2007).

According to Cando et al. (2014), the total phenolic concentrations in *E. angustifolium* were reported to range from 12.20 to 65.35 mg gaE per dry sample, and these results were found to be consistent with previous studies employing similar extraction procedures (Kähkönen et al. 1999; Wojdyło et al. 2007). In line with our findings, it has been reported that *E. angustifolium* possesses high phenolic concentrations compared to other herbs and medicinal plants (Kähkönen et al. 1999). However, the results obtained in the studies were influenced by the extraction method and the solvent used for extraction. Specifically, Kähkönen et al. (1999) reported 32.2 and 27.59 mg gaE/g dry sample results for studies using 80% methanol and 75% of the same solvent, respectively. Using the same procedure, Wojdyło et al. (2007) reported concentrations of phenolic compounds in *E. angustifolium* samples (4 × 10–3 mg gaE/g dry sample) that were 1000 times lower.

According to the study by Alizadeh Behbahani et al. (2024), the combination of *Satureja intermedia* and *Ducrosia anethifolia* essential oils has been reported to exhibit a stronger antioxidant effect against free radicals compared to the essential oils used individually. Similarly, the *E. angustifolium* extract evaluated in our study reached a DPPH radical scavenging capacity of 48.80% ± 3.74%, indicating a significant antioxidant capacity when compared. According to the data by Noshad et al. (2023a), the TPC of *Coriandrum sativum* seeds and *Cuminum cyminum* essential oils was reported as 76.52 ± 0.83 mg GAE/g and 44.28 ± 0.66 mg GAE/g, respectively. In comparison, the TFC was recorded as 34.5 ± 0.61 mg QE/g and 22.6 ± 0.74 mg QE/g, respectively. According to our findings, it has been reported that *E. angustifolium* has higher phenolic and flavonoid content compared to *Coriandrum sativum* seeds and *Cuminum cyminum* essential oils. According to the results of Jalil Sarghaleh et al. (2023), the TPC of *Prangos ferulacea* extract was reported as 202.04 ± 5.46 mg GAE/g, and the TFC was determined to be 1909.46 ± 13 μg QE/g. The antioxidant activity of the extract was evaluated using the DPPH test, and the IC_50_ value was found to be 274 ± 7.2 μg/mL. Additionally, the FRAP test assessed the antioxidant activity, and the IC_50_ value was reported as 1.92 ± 0.05 μg AAE/g. These results demonstrate that the extract has high antioxidant properties. We found that *E. angustifolium* has higher phenolic and flavonoid content than *Prangos ferulacea* extract. Additionally, it demonstrates good antioxidant activity. According to the data of Noshad et al. (2022), the antioxidant activity of *Citrus bergamia* essential oil evaluated using the DPPH test was found to be 212 ± 2.65 μg/mL. In our study, using the same method, the antioxidant activity of *E. angustifolium* extract was measured as 48.80% ± 3.74%, demonstrating good antioxidant activity. In the study conducted by Alizadeh Behbahani et al. (2025), the TPC values for *Nepeta menthoides* (NM), *Nepeta cephalotes* (NC), and the combined NM/NC essential oils were determined to be 106.90, 126.50, and 133.40 mg GAE/g, respectively. Correspondingly, the TFC values for these oils were measured as 45.52, 51.40, and 53.60 mg QE/g. The findings revealed that the combined NM/NC essential oil exhibited stronger antioxidant activity in the DPPH test than the individual essential oils. Alizadeh Behbahani et al. (2025) found that the NM/NC blend had stronger antioxidant capacity, while *E. angustifolium* showed good antioxidant potential but exhibited slightly lower activity in the DPPH and FRAP tests. However, its high TPC and TFC values suggest it is still a potent antioxidant source.

### 4.2 Molecular Docking Studies

The experimental results indicate that the primary effect of *E. angustifolium* extract is to reduce lipid oxidation. The TBARS analysis showed a significant reduction in lipid oxidation in the groups with the extract. Lipoxygenase (LOX), one of the key enzymes that initiates lipid peroxidation by oxidizing polyunsaturated fatty acids, may be inhibited by the extract due to its high antioxidant capacity (as indicated by the DPPH and FRAP values), thereby slowing the oxidative process. Additionally, since the oxidative effect of LOX can lead to color changes, preserving color stability in the extract‐treated samples may be associated with inhibiting this enzyme. Maintaining pH balance during storage also suggests a delay in oxidative deterioration. Therefore, LOX has been identified as the main target in the docking study to understand better the fundamental mechanism that reduces lipid oxidation.

Three peptides isolated from canned meat have been found to exhibit significant inhibitory activity: RPPPPPPPPAD for DPP‐IV inhibition, ARPPPGPPPLGPPPPGP for ACE‐I inhibition, and PPGPPPPP for α‐glucosidase inhibition (Wójciak and Kęska 2023). In silico bioinformatics analyses revealed the interaction of the selected peptides with the 2QT9, 1O86, and 5NN8 protein receptors using molecular docking. The optimal binding regions were determined through these analyses, with binding energies of −8.4 kcal/mol for the 5NN8 receptor, −9.6 kcal/mol for the 1O86 receptor, and −9.1 kcal/mol for the 2QT9 receptor. These findings suggest that the peptides could be useful for designing functional foods, contributing to consumer health protection (Wójciak and Kęska 2023). In our study, the chemical components of the *E. angustifolium* extract in beef burgers were identified through HPLC and GC–MS analyses. The main components of the extract include β‐Tujon (−6.3), α‐Pinen (−5.8), ascorbic acid (−5.1), and gallic acid (−6.8), and the interaction of these compounds with the lipoxygenase enzyme was demonstrated through molecular docking analyses, showing moderate binding energies and confirming their lipoxygenase inhibitory properties in silico.

Additionally, in the study by Mosallaie et al. (2024), it was reported that ferulic acid, one of the chemical components of *Convolvulus arvensis* extract, exhibits a binding energy of −6.2 kcal/mol with the target enzyme, indicating a strong interaction and the potential to modulate glutathione peroxidases. For *E. angustifolium* extract, the binding energies of its chemical components with the protein 9R‐lipoxygenase (5EK8) receptor were determined as follows: beta‐THUJONE −6.3 kcal/mol, alpha‐PINENE −5.8 kcal/mol, Ascorbic acid −5.1 kcal/mol, and Gallic acid −6.8 kcal/mol. These data suggest that the components of *E. angustifolium* may play an important role in modulating the 9R‐lipoxygenase (5EK8) receptor.

### 4.3 pH Values of Beef Burgers

The pH values of the beef burgers during the storage period showed no significant effect from the plant extract treatment on the first day. However, on Days 4 and 8, pH values decreased in the treatment groups, particularly in T2 and T3 (*p* < 0.05). Over the storage period, the pH decreased in all groups except for T1. Specifically, the pH in the control group decreased from 5.62 to 5.17, from 5.51 to 5.06 in T2, and from 5.64 to 5.00 in T3. Ferysiuk et al. (2022b) stated that canned meat samples containing 50, 100, and 150 ppm extract had significantly (*p* < 0.05) lower pH values compared to the control group and those containing 1000 ppm extract (6.53 and 6.56, respectively). The authors mentioned that the lower pH values persisted for 60 days of storage and then began to rise and decrease again. Zhou et al. (2020) suggested that the pH decrease in the samples is associated with the presence of acidic compounds such as phenolic acids in the plant extract.

### 4.4 Color Properties of Beef Burgers

The * value (redness) is a key color parameter in evaluating meat oxidation, as a reduction in the desired bright red color in meat products makes them unacceptable to consumers (Renerre 1999). Fresh beef's appealing bright red color is due to oxymyoglobin (OxyMb) (Faustman et al. 2010). The color change in meat during chilled storage is primarily characterized by a loss of redness, generally linked to the accumulation of metmyoglobin (MetMb) on the meat's surface, contrary to the results we obtained. According to our results, on Days 1 and 8, the use of 9 g extract negatively affected the a* value, while the application of 1 and 3 g plant extract obtained from *E. angustifolium* improved the a* value, suggesting that these concentrations could be recommended for extending shelf life and maintaining the desired bright red color.

Upon reviewing the TBARS values presented in Table 4, it was observed that on Days 1 and 4, the plant extracts at 1 and 3 g exhibited antioxidant effects on meat oxidation. In contrast, 9 g plant extract displayed pro‐oxidant characteristics, resulting in a higher TBARS number than the control group. The accumulation of MetMb during chilled storage and, consequently, the discolouration of meat largely depends on the presence of reducing systems in the meat and lipid oxidation (Faustman et al. 2010). Primary lipid oxidation products such as hydroperoxides and other reactive oxygen species (ROS) are known to oxidize the ferrous iron (Fe^2+^) in OxyMb into its ferric form (Fe^3+^) in MetMb (Faustman et al. 2010). Several studies have investigated the effects of different antioxidants on meat and meat products' color and concluded that meat oxidation decreases a* values (Lee, Hendricks, et al. 1998; Lee, Mbwambo, et al. 1998; Yoo et al. 2004). Akamittath et al. (1990) suggested that color fading or the formation of oxidized pigments might promote lipid oxidation. However, O'Grady et al. (2000) proposed that oxymyoglobin oxidation followed lipid oxidation in minced beef. Nevertheless, many researchers have attempted to establish a correlation between lipid oxidation and discolouration in meat products (Akamittath et al. 1990; Gray et al. 1996). Similarly, we observed that the protective effects of the plant extract against lipid oxidation also influenced the increase in a* value. However, by the 8th day, the positive effects on lipid oxidation regarding color stabilization were not observed across all concentrations. Some studies supporting our findings have reported no interaction between lipid oxidation and myoglobin oxidation, indicating that some natural extracts, despite containing polyphenolic compounds, may slow down lipid oxidation without affecting the discolouration of the meat (McBride et al. 2007). This aligns with our results; although natural antioxidants inhibited lipid oxidation in ground beef, their ability to stabilize color was inconsistent across all concentrations.

Similarly, in a study where willow herb ( *Epilobium hirsutum* L.) extract was added to beef patties at concentrations of 50, 200, and 800 ppm, it was reported that the addition of the extract at 800 ppm resulted in more intense discolouration, which contradicted the antioxidant effects of this phenolic‐rich extract on lipids (Cando et al. 2014). Likewise, several studies highlight the absence of a link between lipid oxidation and OxyMb oxidation in meat systems. McBride et al. (2007) reported that rosemary extracts reduced lipid oxidation but had no effect on preserving redness in fresh ground beef. Another study found that adding sesamol reduced lipid oxidation in pork and beef but enhanced OxyMb oxidation (Hayes et al. 2009). In line with our findings, these authors reported dose‐dependent pro‐oxidant effects of the phenolic compound on OxyMb oxidation. Hayes et al. (2009) attributed sesamol's ability to promote OxyMb oxidation to the pro‐oxidant properties of these phenolic compounds' quinone and catechol forms. These oxidized plant phenolics have been described as accelerators of OxyMb oxidation (Castro et al. 1977). The formation of quinone forms of plant phenolics is associated with the presence and consumption of oxygen, and this oxygen consumption process is linked to browning reactions such as Maillard and enzymatic reactions, where transition metals like iron play a significant role. It has also been reported that a low oxygen concentration in the meat system can promote the formation of MetMb, thus accelerating meat discolouration (Faustman et al. 2010).

### 4.5 TBARS Numbers of Beef Burgers

Table 4 presents the TBARS values of beef burgers prepared with *E. angustifolium* (fireweed) extract during an 8‐day storage period. During the initial storage days, TBARS values ranged from 0.197 to 0.830 mg MA/kg, increasing to 0.710–0.931 mg MA/kg on day 8. The T3 group exhibited higher TBARS values than other treatments, indicating that adding 9 g *E. angustifolium* caused an oxidant effect during the storage period. However, this effect dissipated on day 8. These findings can be partly explained by the ability of these extracts to exhibit both prooxidant and antioxidant activities under certain conditions. Similarly, this phenomenon was close to the findings of Ünal et al. (2014), who highlighted that high concentrations of some plants act as prooxidants, leading to tissue damage because of the formation of harmful phenoxyl radicals. Ferysiuk et al. (2022a) also mentioned that using *E. angustifolium* extract at higher levels could present reverse effects due to the antioxidant and pro‐oxidant balance disruption. Therefore, Rey et al. (2005) reported that its addition at a low concentration indicated an inhibitory activity on lipid oxidation in pork patties. The results of their study explain a comparable dose‐dependent behavior in preventing the oxidation rate of cooked pork patties to that observed for *E. angustifolium* extract. The oxidation‐inhibiting ability clearly depended on the amount of extract added. In our study, all beef burgers treated with *E. angustifolium* indicated lower TBARS numbers at the end of the storage. This can be attributed to a rich source of phenolic compounds of *E. angustifolium* . *E. angustifolium* phenolic acids and flavonoids (such as gallic acid and epicatechin) were the main constituents to prevent lipid oxidation. Deng et al. (2018) stated that the influence of phenolic acids may not only depend on their scavenging reactive oxygen species but also inhibit their generation. It is well known that their antioxidant activities were mainly linked with the TPC. In our current study, the DPPH, FRAP, TPC, and TFC of *E. angustifolium* extract were found to be 48.80% ± 3.74%, 2198.05 ± 78.56 mg/L, 1263.48 ± 12.13 mg GAE/L, and 278.43 ± 3.27 mg ce/L, respectively (Table 2).

## 5 Conclusions

The high total phenolic and flavonoid contents of *E. angustifolium* extract, along with its significant antioxidant activity, strongly highlight the antioxidant properties of this plant. Adding the extract to meat products led to noticeable changes in the pH and color parameters of the meat, with color changes observed, particularly at the higher concentration of 9 g, where prooxidant effects were detected. Lipid oxidation was more clearly observed through TBARS analysis, with increased oxidation occurring at higher extract concentrations. Phytochemical analyses confirmed the presence of bioactive compounds such as gallic acid, α‐pinene, and β‐thujone, and these compounds exhibited moderate binding affinities in molecular docking analyses. These findings suggest that *E. angustifolium* extract, particularly when used at appropriate concentrations, holds significant potential for preventing oxidation and preserving the quality of meat products.

## Author Contributions

**Nazik Meziyet Dilek:** methodology, writing – original draft, data curation, data analysis; **Abidin Gümrükçüoğlu:** methodology, data analysis; **Gamze Demirel:** research; **Alper Durmaz:** material provision, writing; **Emine Incilay Torunoğlu:** writing – review and editing, data curation; **Erdi Can Aytar:** visualization, software, formal analysis, conceptualisation, writing – review and editing, supervision, methodology, data curation; **Kübra Ünal:** methodology, writing – original draft, data curation, data analysis.

## Conflicts of Interest

The authors declare no conflicts of interest.

## Data Availability

This published article includes all the datasets generated or analyzed during this study.

## References

[fsn370125-bib-0001] Akamittath, J. G. , C. J. Brekke , and E. G. Schanus . 1990. “Lipid Oxidation and Color Stability in Restructured Meat Systems During Frozen Storage.” Journal of Food Science 55: 1513–1517. 10.1111/J.1365-2621.1990.TB03557.X.

[fsn370125-bib-0002] Akbulut, İ. , E. Gürbüz , A. Rayman Ergün , and T. Baysal . 2021. “Ginkgo Biloba Bitki Ekstraktı ile Muamele Edilmiş Kayısıların Yapay Yolla Kurutulması ve Kalite Özelliklerinin Belirlenmesi.” Journal of Advanced Research in Natural and Applied Sciences 7, no. 1: 145–159. 10.28979/jarnas.840237.

[fsn370125-bib-0003] Aktas, R. N. , and I. Tontul . 2021. “Usability of Soapwort and Horse Chestnut Saponin Extracts as Foaming Agents in Foam Mat Drying of Pomegranate Juice.” Journal of the Science of Food and Agriculture 101: 786–793. 10.1002/JSFA.10770. 32869316

[fsn370125-bib-0004] Alan, Y. 2023. “Chemical Changes of Potential Probiotic Lactiplantibacillus Plantarum and *Lactobacillus pentosus* Starter Cultures in Natural Gemlik Type Black Olive Fermentation.” Food Chemistry 434: 137472. 10.1016/j.foodchem.2023.137472. 37722330

[fsn370125-bib-0005] Alizadeh Behbahani, B. , M. Noshad , F. Falah , F. Zargari , Z. Nikfarjam , and A. Vasiee . 2024. “Synergistic Activity of Satureja Intermedia and Ducrosia Anethifolia Essential Oils and Their Interaction Against Foodborne Pathogens: A Multi‐Ligand Molecular Docking Simulation.” LWT 205: 116487. 10.1016/J.LWT.2024.116487.

[fsn370125-bib-0006] Alizadeh Behbahani, B. , M. Noshad , F. Falah , F. Zargari , Z. Nikfarjam , and A. Vasiee . 2025. “First Report on the Synergy of Nepeta Menthoides and Nepeta Cephalotes Essential Oils for Antimicrobial and Preservation Applications: A Multi‐Ligand Molecular Docking Simulation.” Applied Food Research 5: 100707. 10.1016/J.AFRES.2025.100707.

[fsn370125-bib-0007] Aytar, E. C. , and B. Aydın . 2024. “Investigation of Chemical Composition, Antioxidant Properties, and Molecular Docking in Different Roasting Stages of Coffee Beans.” Food and Bioprocess Technology 18, no. 2: 1464–1482. 10.1007/S11947-024-03539-1.

[fsn370125-bib-0008] Babaoğlu, A. S. , K. Unal , N. M. Dilek , H. B. Poçan , and M. Karakaya . 2022. “Antioxidant and Antimicrobial Effects of Blackberry, Black Chokeberry, Blueberry, and Red Currant Pomace Extracts on Beef Patties Subject to Refrigerated Storage.” Meat Science 187: 108765. 10.1016/J.MEATSCI.2022.108765. 35183845

[fsn370125-bib-0009] Biovia, D. S. , and D. Systèmes . 2016. Biovia, Discovery Studio Modeling Environment. Dassault Systèmes Biovia.

[fsn370125-bib-0010] BizimBitkiler . 2025. “Türkiye Bitkileri Listesi.” Nezahat Gökyiğit Botanik Bahçesi, 2019. https://bizimbitkiler.org.tr/yeni/demos/technical/.

[fsn370125-bib-0011] Cando, D. , D. Morcuende , M. Utrera , and M. Estévez . 2014. “Phenolic‐Rich Extracts From Willowherb ( *Epilobium hirsutum* L.) Inhibit Lipid Oxidation but Accelerate Protein Carbonylation and Discoloration of Beef Patties.” European Food Research and Technology 238: 741–751. 10.1007/S00217-014-2152-9.

[fsn370125-bib-0012] Castro, C. E. , G. M. Hathaway , and R. Havlin . 1977. “Oxidation and Reduction of Iron Porphyrins and Hemoproteins by Quinones and Hydroquinones.” Journal of the American Chemical Society 99: 8032–8039. 10.1021/JA00466A042.

[fsn370125-bib-0013] Chen, G. , and H. Chen . 2011. “Extraction and Deglycosylation of Flavonoids From Sumac Fruits Using Steam Explosion.” Food Chemistry 126: 1934–1938. 10.1016/J.FOODCHEM.2010.12.025. 25213979

[fsn370125-bib-0014] Davis, P. 1972. Flora of Turkey and the East Aegean Islands. Vol. 4. Edinburgh University Press.

[fsn370125-bib-0015] Deng, L. Q. , S. Y. Zhou , J. X. Mao , et al. 2018. “HPLC‐ESI‐MS/MS Analysis of Phenolics and In Vitro Antioxidant Activity of *Epilobium angustifolium* L.” Natural Product Research 32: 1432–1435. 10.1080/14786419.2017.1344659. 28637366

[fsn370125-bib-0016] Faustman, C. , Q. Sun , R. Mancini , and S. P. Suman . 2010. “Myoglobin and Lipid Oxidation Interactions: Mechanistic Bases and Control.” Meat Science 86: 86–94. 10.1016/J.MEATSCI.2010.04.025. 20554121

[fsn370125-bib-0017] Ferysiuk, K. , K. M. Wójciak , and P. Kęska . 2022a. “Effect of Willow Herb ( *Epilobium angustifolium* L.) Extract Addition to Canned Meat With Reduced Amount of Nitrite on the Antioxidant and Other Activities of Peptides.” Food & Function 13: 3526–3539. 10.1039/D1FO01534F. 35253026

[fsn370125-bib-0018] Ferysiuk, K. , K. M. Wójciak , and M. Trząskowska . 2022b. “Fortification of Low‐Nitrite Canned Pork With Willow Herb (*Epilobium angustifolium* L.).” International Journal of Food Science & Technology 57, no. 7: 4194–4210. 10.1111/IJFS.15739.

[fsn370125-bib-0019] Filipiak‐Szok, A. , M. Kurzawa , and E. Szłyk . 2015. “Determination of Toxic Metals by ICP‐MS in Asiatic and European Medicinal Plants and Dietary Supplements.” Journal of Trace Elements in Medicine and Biology 30: 54–58. 10.1016/j.jtemb.2014.10.008. 25467854

[fsn370125-bib-0020] Gray, J. I. , E. A. Gomaa , and D. J. Buckley . 1996. “Oxidative Quality and Shelf Life of Meats.” Meat Science 43: 111–123. 10.1016/0309-1740(96)00059-9. 22060645

[fsn370125-bib-0021] Güven, S. , S. Makbul , F. Mertayak , and K. Coşkunçelebi . 2021. “Anatomical Properties of Epilobium and Chamaenerion From a Taxonomical Perspective in Turkey.” Protoplasma 258: 827–847. 10.1007/S00709-020-01602-0. 33507396

[fsn370125-bib-0022] Hayes, J. E. , V. Stepanyan , P. Allen , M. N. O'Grady , N. M. O'Brien , and J. P. Kerry . 2009. “The Effect of Lutein, Sesamol, Ellagic Acid and Olive Leaf Extract on Lipid Oxidation and Oxymyoglobin Oxidation in Bovine and Porcine Muscle Model Systems.” Meat Science 83, no. 2: 201–208. 10.1016/J.MEATSCI.2009.04.019. 20416759

[fsn370125-bib-0023] Jalil Sarghaleh, S. , B. Alizadeh Behbahani , M. Hojjati , A. Vasiee , and M. Noshad . 2023. “Evaluation of the Constituent Compounds, Antioxidant, Anticancer, and Antimicrobial Potential of Prangos Ferulacea Plant Extract and Its Effect on *Listeria monocytogenes* Virulence Gene Expression.” Frontiers in Microbiology 14: 1202228. 10.3389/FMICB.2023.1202228. 37492261 PMC10364450

[fsn370125-bib-0024] Kadam, P. , M. Patil , and K. Yadav . 2018. “A Review on Phytopharmacopial Potential of *Epilobium angustifolium* .” Pharmacognosy Journal 10, no. 6: 1076–1078. 10.5530/pj.2018.6.181.

[fsn370125-bib-0025] Kähkönen, M. P. , A. I. Hopia , H. J. Vuorela , et al. 1999. “Antioxidant Activity of Plant Extracts Containing Phenolic Compounds.” Journal of Agricultural and Food Chemistry 47: 3954–3962. 10.1021/JF990146L. 10552749

[fsn370125-bib-0026] Kavaz Yüksel, A. , E. Dikici , M. Yüksel , M. Işık , F. Tozoğlu , and E. Köksal . 2021. “Phytochemical, Phenolic Profile, Antioxidant, Anticholinergic and Antibacterial Properties of *Epilobium angustifolium* (Onagraceae).” Journal of Food Measurement and Characterization 15: 4858–4867. 10.1007/s11694-021-01050-1.

[fsn370125-bib-0027] Kumar, A. , V. K. Varshney , M. S. M. Rawat , J. R. Martinez , and E. E. Stashenko . 2018. “HS‐SPME/GC/GC‐MS Analysis of Volatile Constituents of Morina Longifolia Wall.” Journal of Essential Oil Bearing Plants 21, no. 1: 155–163. 10.1080/0972060X.2018.1437782.

[fsn370125-bib-0028] Lambooij, E. , C. M. Potgieter , C. M. Britz , G. L. Nortje , and C. Pieterse . 1999. “Effects of Electrical and Mechanical Stunning Methods on Meat Quality in Ostriches.” Meat Science 52, no. 3: 331–337. 22062583 10.1016/s0309-1740(99)00010-8

[fsn370125-bib-0029] Lee, B. J. , D. G. Hendricks , and D. P. Cornforth . 1998a. “Antioxidant Effects of Carnosine and Phytic Acid in a Model Beef System.” Journal of Food Science 63: 394–398. 10.1111/J.1365-2621.1998.TB15750.X.

[fsn370125-bib-0030] Lee, S. K. , Z. H. Mbwambo , H. Chung , et al. 1998b. “Evaluation of the Antioxidant Potential of Natural Products.” Combinatorial Chemistry & High Throughput Screening 1: 35–46. 10.2174/138620730101220118151526. 10499128

[fsn370125-bib-0031] Manessis, G. , A. I. Kalogianni , T. Lazou , M. Moschovas , I. Bossis , and A. I. Gelasakis . 2020. “Plant‐Derived Natural Antioxidants in Meat and Meat Products.” Antioxidants 9: 1215. 10.3390/ANTIOX9121215. 33276503 PMC7761563

[fsn370125-bib-0032] McBride, N. T. M. , S. A. Hogan , and J. P. Kerry . 2007. “Comparative Addition of Rosemary Extract and Additives on Sensory and Antioxidant Properties of Retail Packaged Beef.” International Journal of Food Science and Technology 42: 1201–1207. 10.1111/J.1365-2621.2006.01342.X.

[fsn370125-bib-0033] Mosallaie, F. , M. Pirnia , Z. Dehghan , et al. 2024. “Unveiling the Chemical Composition, Antioxidant and Antibacterial Properties, and Mechanistic Insights of *Convolvulus arvensis* Extract Through Molecular Docking Simulations.” Applied Food Research 4: 100580. 10.1016/J.AFRES.2024.100580.

[fsn370125-bib-0034] Myerscough, P. J. 1980. “ *Epilobium angustifolium* L.” Journal of Ecology 68: 1047. 10.2307/2259474.

[fsn370125-bib-0035] Noshad, M. , B. Alizadeh Behbahani , and Z. Nikfarjam . 2022. “Chemical Composition, Antibacterial Activity, and Antioxidant Activity of *Citrus bergamia* Essential Oil: Molecular Docking Simulations.” Food Bioscience 50: 102123. 10.1016/J.FBIO.2022.102123.

[fsn370125-bib-0036] Noshad, M. , B. A. Behbahani , Z. Nikfarjam , and F. Zargari . 2023a. “Antimicrobial Activity Between *Coriandrum sativum* Seed and *Cuminum cyminum* Essential Oils Against Foodborne Pathogens: A Multi‐Ligand Molecular Docking Simulation.” LWT 185: 115217. 10.1016/J.LWT.2023.115217.

[fsn370125-bib-0037] Noshad, M. , B. A. Behbahani , Z. Nikfarjam , F. Zargari , and J. Simal‐Gandara . 2023b. “Perception Into the Binding of Soy Protein Isolate With Essential Oils Using Multispectroscopic and QuickVina‐W.” LWT 185: 115157. 10.1016/J.LWT.2023.115157.

[fsn370125-bib-0038] Nowak, A. , J. Zielonka‐Brzezicka , M. Perużyńska , and A. Klimowicz . 2022. “ *Epilobium angustifolium* L. as a Potential Herbal Component of Topical Products for Skin Care and Treatment—A Review.” Molecules 27, no. 11: 3536. 10.3390/molecules27113536. 35684473 PMC9182203

[fsn370125-bib-0039] O'Grady, M. N. , F. J. Monahan , R. M. Burke , and P. Allen . 2000. “The Effect of Oxygen Level and Exogenous α‐Tocopherol on the Oxidative Stability of Minced Beef in Modified Atmosphere Packs.” Meat Science 55: 39–45. 10.1016/S0309-1740(99)00123-0. 22060902

[fsn370125-bib-0040] Petcu, C. D. , D. Tăpăloagă , O. D. Mihai , et al. 2023. “Harnessing Natural Antioxidants for Enhancing Food Shelf Life: Exploring Sources and Applications in the Food Industry.” Food 3176, no. 12: 3176. 10.3390/FOODS12173176. PMC10486681 37685108

[fsn370125-bib-0041] POWO . 2025a. “*Epilobium* Dill. ex L.” Plants of the World Online. https://powo.science.kew.org/taxon/urn:lsid:ipni.org:names:30000954‐2.

[fsn370125-bib-0042] POWO . 2025b. “*Epilobium angustifolium* L.” Plants of the World Online, Kew Science. https://powo.science.kew.org/taxon/urn:lsid:ipni.org:names:92411‐2.

[fsn370125-bib-0043] Renerre, M. 1999. “Biochemical Basis of Fresh Meat Colour.” In *Proceedings of the 45th International Congress of Meat Science and Technology*, AGRIS—International System for Agricultural Science and Technology.

[fsn370125-bib-0044] Rey, A. I. , A. Hopia , R. Kivikari , and M. Kahkonen . 2005. “Use of Natural Food/Plant Extracts: Cloudberry (*Rubus chamaemorus*), Beetroot (*Beta vulgaris*) or Willow Herb (*Epilobium angustifolium*) to Reduce Lipid Oxidation of Cooked Pork Patties.” LWT‐Food Science and Technology 38: 363–370. 10.1016/J.LWT.2004.06.010.

[fsn370125-bib-0045] Schepetkin, I. A. , A. G. Ramstead , L. N. Kirpotina , J. M. Voyich , M. A. Jutila , and M. T. Quinn . 2016. “Therapeutic Potential of Polyphenols From *Epilobium angustifolium* (Fireweed).” Phytotherapy Research 30: 1287–1297. 10.1002/ptr.5648. 27215200 PMC5045895

[fsn370125-bib-0046] Seal, T. 2016. “Quantitative HPLC Analysis of Phenolic Acids, Flavonoids and Ascorbic Acid in Four Different Solvent Extracts of Two Wild Edible Leaves, Sonchus Arvensis and Oenanthe Linearis.” Journal of Applied Pharmaceutical Science 6, no. 2: 157–166. 10.7324/JAPS.2016.60225.

[fsn370125-bib-0047] Shikov, A. N. , E. A. Poltanov , H. J. D. Dorman , V. G. Makarov , V. P. Tikhonov , and R. Hiltunen . 2006. “Chemical Composition and In Vitro Antioxidant Evaluation of Commercial Water‐Soluble Willow Herb (*Epilobium angustifolium* L.) Extracts.” Journal of Agricultural and Food Chemistry 54, no. 10: 3617–3624. 10.1021/JF052606I. 19127734

[fsn370125-bib-0048] Tarladgis, B. G. , B. M. Watts , M. T. Younathan , and L. Dugan . 1960. “A Distillation Method for the Quantitative Determination of Malonaldehyde in Rancid Foods.” Journal of the American Oil Chemists' Society 37, no. 1: 44–48. 10.1007/BF02630824.

[fsn370125-bib-0049] Ünal, K. , A. S. Babaoglu , and M. Karakaya . 2014. “Effect of Oregano, Sage and Rosemary Essential Oils on Lipid Oxidation and Color Properties of Minced Beef During Refrigerated Storage.” Journal of Essential Oil‐Bearing Plants 17: 797–805. 10.1080/0972060X.2014.956803.

[fsn370125-bib-0050] Uysal, A. , G. Zengin , Y. Durak , and A. Aktumsek . 2016. “Centaurea Pterocaula Özütlerinin Antioksidan ve Antimutajenik Özellikleri Ile Enzim Inhibitör Potansiyellerinin Incelenmesi.” Marmara Pharmaceutcal Journal 20, no. 3: 232–242. 10.12991/mpj.20162094922.

[fsn370125-bib-0051] Wang, W. , P. Xiong , H. Zhang , Q. Zhu , C. Liao , and G. Jiang . 2021. “Analysis, Occurrence, Toxicity, and Environmental Health Risks of Synthetic Phenolic Antioxidants: A Review.” Environmental Research 201: 111531. 10.1016/j.envres.2021.111531. 34146526

[fsn370125-bib-0052] Wójciak, K. M. , and P. Kęska . 2023. “Biological Activity of Canned Pork Meat Fortified Black Currant Leaf Extract: In Vitro, In Silico, and Molecular Docking Study.” Molecules 28: 8009. 10.3390/MOLECULES28248009. 38138499 PMC10745298

[fsn370125-bib-0053] Wojdyło, A. , J. Oszmiański , and R. Czemerys . 2007. “Antioxidant Activity and Phenolic Compounds in 32 Selected Herbs.” Food Chemistry 105: 940–949. 10.1016/J.FOODCHEM.2007.04.038.

[fsn370125-bib-0054] Yoo, K. M. , K. W. Lee , J. B. Park , H. J. Lee , and I. K. Hwang . 2004. “Variation in Major Antioxidants and Total Antioxidant Activity of Yuzu (Citrus Junos Sieb ex Tanaka) During Maturation and Between Cultivars.” Journal of Agricultural and Food Chemistry 52: 5907–5913. 10.1021/JF0498158. 15366841

[fsn370125-bib-0055] Zhang, N. , T. Chen , S. Ye , S. Gao , and Y. Dong . 2022. “Comparative Analysis With GC–MS of Fatty Acids and Volatile Compounds of *Taraxacum kok‐saghyz* Rodin and *Taraxacum officinale* as Edible Resource Plants.” Separations 9, no. 10: 314. 10.3390/separations9100314.

[fsn370125-bib-0056] Zhou, Y. , Q. Y. Wang , and S. Wang . 2020. “Effects of Rosemary Extract, Grape Seed Extract and Green Tea Polyphenol on the Formation of N‐Nitrosamines and Quality of Western‐Style Smoked Sausage.” Journal of Food Processing & Preservation 44, no. 6: e14459. 10.1111/JFPP.14459.

